# Melatonin as an Add-On Treatment of COVID-19 Infection: Current Status

**DOI:** 10.3390/diseases9030064

**Published:** 2021-09-20

**Authors:** Gregory M. Brown, Seithikurippu R. Pandi-Perumal, Harold Pupko, James L. Kennedy, Daniel P. Cardinali

**Affiliations:** 1 Centre for Addiction and Mental Health, Molecular Brain Sciences, Department of Psychiatry, University of Toronto, Toronto, ON M5T 1R8, Canada; jim.kennedy@camh.ca; 2 Somnogen Canada Inc., Toronto, ON M6H 1C5, Canada; pandiperumal2021@gmail.com; 3 Saveetha Institute of Medical and Technical Sciences, Saveetha Medical College and Hospitals, Saveetha University, Chennai 600077, India; 4 Primary Care Mental Health Physician, Bathurst St., Toronto, ON M3H 3S3, Canada; Pupko@Rogers.com; 5 Faculty of Medical Sciences, Pontificia Universidad Católica Argentina, Buenos Aires 1007, Argentina; cardinalidanielpedro@gmail.com

**Keywords:** melatonin, COVID-19, RCT, immunity, cytokines, mitochondria, antioxidant

## Abstract

This brief review was written to provide a perspective on the flurry of reports suggesting that melatonin can be an important add-on therapy for COVID-19. Despite the passage of more than 60 years since its discovery and much evidence representing the contrary, there has been great reluctance to conceive melatonin as anything other than a hormone. Many other body chemicals are known to have multiple roles. Melatonin was first shown to be a hormone derived from the pineal gland, to be actively synthesized there only at night, and to act on targets directly or via the G-protein-coupled receptors (GPCRs) superfamily. It is of note that over 40 years ago, it was also established that melatonin is present, synthesized locally, and acts within the gastrointestinal tract. A wider distribution was then found, including the retina and multiple body tissues. In addition, melatonin is now known to have non-hormonal actions, acting as a free radical scavenger, an antioxidant, and as modulating immunity, dampening down innate tissue responses to invaders while boosting the production of antibodies against them. These actions make it a potentially excellent weapon against infection by the SARS-CoV-2 virus. Early published results support that thesis. Recently, a randomized controlled study reported that low doses of melatonin significantly improved symptoms in hospitalized COVID-19 patients, leading to more rapid discharge with no side effects, while significantly decreasing levels of CRP, proinflammatory cytokines, and modulating dysregulated genes governing cellular and humoral immunity. It is now critical that these trials be repeated, with dose-response studies conducted and safety proven. Numerous randomized controlled trials are ongoing, which should complete those objectives while also allowing for a more thorough evaluation of the mechanisms of action and possible applications to other severe diseases.

## 1. Introduction

Melatonin’s original discovery is well known. Seeking a skin-lightening agent, Aaron Lerner, a dermatologist, together with his coworkers, isolated melatonin from beef pineal glands, measuring its activity in frog melanocytes [1]. However, administration of one gram per day orally in five hyperpigmented patients showed minimal skin-lightening in only one patient. The only notable effect was drowsiness together with an apparent minor decrease in serum-luteinizing hormone levels [2]. Further clinical studies have focused almost exclusively on its role as a hormone. With the development of immunoassays, studies showed that it was secreted by the pineal gland under control by the body’s master clock, the hypothalamic suprachiasmatic nuclei, and that light and especially blue light would suppress secretion [3]. The most evident hormonal action of melatonin was its effects on the body circadian rhythm: oral administration during the morning shortly after waking led to a delay in body rhythms the next day, while administration prior to going to bed led to a rhythm advance. This effect was followed by its use in the delayed-phase sleep disorder and jet lag, often in combination with controlled lighting, which has effects on rhythms that are the inverse of melatonin [4].

In the early 70s, a Russian group found melatonin in the human appendix by bioassay and speculated that there might be local synthesis, supporting the findings with chromatography [5,6]. George Bubenik followed up that lead using immunohistology with a highly specific antiserum validated by gas chromatography spectrometry [7] and localized it in the gastrointestinal tract (GIT) in areas corresponding to enterochromaffin cells. Melatonin extended from the duodenum to the rectum. In a series of studies, Bubenik’s group established that GIT melatonin did not derive from the pineal gland as levels were not altered by pinealectomy, considering the total GIT content was some 600 to 800-folds greater than the pineal content, and levels within the GIT changed with food intake but displayed no day–night difference [8,9]. Notably, in numerous studies, melatonin has been found much more widely in the body using several complementary techniques [10]. There is evidence for local synthesis in many of these tissues [11,12,13,14,15]. There is also rich literature reporting that melatonin is present in all living aerobic entities, including plants and insects [16,17].

It should also be noted that melatonin is highly pleiotropic and as it is amphiphilic, it can diffuse through cell walls or barriers such as that between the blood and brain. Melatonin is a very potent antioxidant. It acts directly rather than through receptors on oxidative/apoptotic signaling [18]. Melatonin prevents DNA damage by oxidation, yields metabolites that are antioxidant, and inhibits pro-oxidative enzymes. It also induces the antioxidant enzymes’ superoxide dismutase while increasing glutathione synthesis [19]. It is noteworthy that mitochondria, the powerhouse of the body that produce most of the body’s free oxygen radicals, are also a major site of melatonin synthesis that can act to neutralize excess free radicals [20]. Mitochondria are especially sensitive to oxidative stress and mutation, leading them to become dysfunctional [21]. Melatonin suppresses proinflammatory cytokine production in M1 macrophages, polarizing them to anti-inflammatory M2 macrophages and thus reducing excess cytokine release [22]. Moreover, melatonin has a two-faced action on immunity, reducing innate immunity and preventing tissue overreaction, while at the same time boosting resistance to external pathogens [23]

## 2. Applicability to COVID-19

There are several mechanisms by which melatonin can interfere directly or indirectly with the binding of the SARS-CoV-2 virus to the target angiotensin-converting enzyme 2 (ACE 2) in tissues that could explain a direct antiviral effect [24,25]. Recently using in vitro experiments in cells, it was demonstrated that melatonin was very effective for prevention control and cross-species transmission mechanisms of animal coronaviruses causing transmissible gastroenteritis, porcine epidemic diarrhea virus, and porcine delta coronavirus [26]. Many other actions of melatonin are also relevant including the role of melatonin in helping synchronize sleep and maintain a normal circadian rhythm [21,25,27,28]. Melatonin plays a major role in the circadian system, regulating the timing of sleep onset so that the circadian rhythm in the synthesis and secretion of pineal melatonin is closely associated with the sleep rhythm in both normal and blind subjects [29]. The onset of nighttime melatonin secretion is initiated approximately 2 h in advance of an individual’s habitual bedtime and has been shown to correlate with the onset of evening sleepiness. Several studies implicate endogenous melatonin in the physiological regulation of the circadian mechanisms governing sleep propensity [30,31]. This chronobiological aspect of melatonin activity is very important because the immune system displays very strong circadian rhythmicity [32]. At the beginning of a daily activity, there is increased expression of proinflammatory mediators such as interleukin (IL)-1β, IL-6, and IL-12, as well as of macrophage and leukocyte activity, which leads to the potential damaging of tissues. By contrast, anti-inflammatory mediators and other growth or angiogenesis factors peak during the resting phase. Both CD4 and CD8 T cells’ activities against viral antigens reach their highest levels during the resting phase, while the cytotoxic activity of natural killer cells is most severe at the beginning of the active part of the day. These T cells that play a key role in the antiviral immune response express both membrane and nuclear melatonin receptors whose activation plays a regulatory role in the response by balancing its various functional arms. In addition, numerous pathways, including both receptor-dependent and independent, have been established by which melatonin may produce neuroprotective effects [18]. For example, the high levels known to exist in the cerebrospinal fluid caused by direct release from the pineal gland could effectively shield the brain from oxidative stress [33].

As for evidence that indicates melatonin helps with COVID-19 disease, there are several animal viral and human septic shock studies in which melatonin has shown potent beneficial effects [21]. In critically affected patients, deep sedation is associated with increased long-term mortality and the administration of melatonin reduces the use of sedation and the frequency of pain, agitation, and anxiety [34], as well as also improves the quality of sleep in intensive care unit patients. Therefore, the rationale for the use of melatonin in COVID-19 illness focuses not only on the attenuation of infection-induced respiratory disorders but also on the general improvement and prevention of possible complications. Concerning the clinical results of melatonin in COVID-19 patients, it must be noted that because of its pharmacokinetic properties, i.e., a very short half-life in the blood, melatonin administration as a fast-release preparation at bedtime may give rise to a chronobiotic signal regardless of the amounts given [35]. Additionally, the sleep regulatory effect of melatonin might have an important, albeit indirect, effect on the immune response because of the well-known importance of sleep on the immune system.

### Studies of Melatonin and COVID-19 Illness

A non-randomized study reported the efficacy of melatonin as an adjuvant in COVID-19. This study determined the efficacy and tolerability of high-dose melatonin (36 mg/day to 72 mg/day p.o. in four divided doses) as an adjuvant therapy in COVID-19 illness [36]. All patients were admitted with chest imaging findings of COVID-19 pneumonia. The 10 patients given melatonin had high-risk features determined for age (>60 years) and/or established comorbidities. Benefits of time for clinical improvement (reduction of symptoms, stabilization and/or regression of lung infiltrates, and decrease in proinflammatory markers) were documented, as well as of a reduction of the need for mechanical ventilation, duration of hospital stay, and outcome (death or recovery and discharge) [36].

Two papers have described the first reported double-blind randomized clinical trial (RCT). That RCT employed the therapeutic algorithm for the use of melatonin in patients with COVID-19, as published by Reiter et al. [37], recommending a daily dose of 3–10 mg p.o. for elderly patients with comorbidities such as sleep disruption. The first paper primarily describes the clinical results in the 44 hospitalized patients with confirmed mild to moderate COVID-19 seen at the Baqiyatallah Hospital in Tehran who completed the study [38]. Patients had been randomly assigned to receive standard of care or standard of care in addition to melatonin at a dose of 3 mg three times daily for 14 days. The 24 patients in the melatonin-treated group exhibited a significant improvement in clinical symptoms (cough, dyspnea, and fatigue) and pulmonary involvement, as well as significantly lower levels of circulating C-reactive protein as compared to 20 controls. Moreover, both the mean time of the hospital discharge of patients and return to baseline health were significantly shorter in the intervention group [38]. The second paper describes a cohort from the same study in which Th1 and Th2-mediated cellular and humoral immunity were evaluated in 40 male and female patients (20 receiving melatonin and 20 controls) [39]. Again, the mean times of recovery and discharge were significantly decreased in the melatonin group and there were no side effects attributable to melatonin. Blood samples were drawn at the beginning and end of treatment. Results, for the first time, significantly established that adjuvant treatment with melatonin controlled and reduced inflammatory cytokines. Moreover, melatonin also significantly controlled and modulated the dysregulated genes governing the humoral and cellular immune systems mediated by Th1 and Th2. Thus, this first randomly controlled RCT strongly indicates that melatonin warrants attention as a treatment adjuvant that has improved both clinical and laboratory findings.

Considering melatonin is a multipotential substance, several different functions of melatonin must be considered in discussing these clinical and cellular observations. Today, it is widely held that the antioxidant properties of melatonin were its original function in primitive organisms. Then, during evolution, melatonin acquired many other more sophisticated and important functions including the synchronization of the organism to the photoperiod and immunomodulating properties in all mammals. The importance of the role of antioxidants is underlined in a clinical study in which the effect of pentoxifylline alone or together with antioxidants (vitamin C, vitamin E, N-acetylcysteine, and melatonin) was evaluated in COVID-19 patients with moderate or severe pneumonia [40]. The antioxidant therapy in addition to the pentoxifylline therapy improved survival scores; lowered lipid peroxidation, IL-6, C-reactive protein, and procalcitonin levels; and increased the systemic total antioxidant capacity and NO_2_- levels as compared to pentoxifylline alone. Unfortunately, no melatonin-alone results were included to allow for comparison.

The RCT study also offers strong support for several clinical observations as, for example, a retrospective analysis at the Columbia University Irving Medical Center, New York, of drugs used to treat respiratory distress in SARS-CoV-2-infected patients who required endotracheal intubation [41]. From the 189,987 patients examined, the authors identified 948 intubation periods across 791 patients who were diagnosed with COVID-19 or were infected with SARS-CoV2 and 3,497 intubation periods across 2,981 patients who were not. Melatonin exposure was significantly associated with a positive outcome in COVID-19 and non-COVID-19 patients. Additionally, melatonin exposure was significantly associated with a positive outcome in COVID-19 patients requiring mechanical ventilation [41].

Interestingly, another recent retrospective analysis showed an association of melatonin with survival in a sample of 2463 COVID-19 patients hospitalized during the first wave of the pandemic, 265 of whom (10.75%) were given 2–6 mg of oral melatonin. The melatonin group exhibited a much lower mortality rate (10.7% vs. 23.7%) compared to the non-melatonin matched group. No differences were found between the two groups in the distribution of CURB-65 scores (a validated scale of clinical severity), indicating that they were similar in terms of illness at admission [42].

## 3. Why Was Melatonin Not Being Routinely Used in COVID-19 Pneumonia?

Medical practice tends to be very conservative when presented with novel methods or treatments, often for good reason. A common response to the suggestion for the use of melatonin to reduce the severity of COVID-19 illness is that melatonin is a hormone and is only involved in sleep regulation. On this note, it must be kept in mind that there are numerous examples of molecules that are similar to melatonin in that they have more than one function. For example, the majority of serotonin is synthesized in enterochromaffin cells of the GIT and has a role in the GIT movement. Nevertheless, it also enters the blood and is taken up in platelets in which it is released during vasoconstriction, and, of course, it is well known as a neurotransmitter with functions in the CNS [43]. Another example is norepinephrine, which is a catecholamine that functions as a neurotransmitter in several brain pathways but is also a hormone synthesized and released into the bloodstream from the adrenal medulla [44].

Three different companies, namely “Servier”,”Takeda”, and “Vanda”, have brought melatonin-related drugs to market: Valdoxan (Agomelatine), Ramelteon (Rozerem), and Hetlioz (Tasimelteon). However, these agents primarily target the G-protein-linked melatonin receptors which do not encompass the full range of melatonin actions that may be involved in the effects reported in COVID-19 [39]. Therefore, further study will be required to determine the precise actions that are responsible for the reported effects on COVID-19. Studying the mechanisms underlying the various documented effects of melatonin in detail may well lead to the discovery of additional patentable chemical entities that will target specific actions beneficial not only for diseases such as COVID-19 but for awider array of inflammatory conditions.

Moving forward, more funding opportunities should be made available from both government and industrial sectors. Additionally, public–private partnerships and academic–industry partnerships could be encouraged, which may transform the research in this direction, providing long-term benefits for all. Hence, these stakeholders should unite and move the field forward. Such fruitful collaboration would offer ideal benefits to the patients and establish the utility of melatonin for multiple medical and mental conditions due to its pleiotropic actions.

Gaps in the knowledge regarding how to use melatonin; limited or lack of knowledge about melatonin and its potentials; widespread false information on the web and on media sources; lack of awareness of the low risk and long-term safety profile of melatonin; and lack of an established standard dosage regimen all can result in the reluctance to give melatonin to patients. Such recalcitrance and knowledge gaps need to be changed rapidly so that melatonin can be used widely as an adjuvant for the treatment of COVID-19. This situation is reminiscent of the Semmelweis discovery concerning that puerperal fever could be prevented by disinfecting hands in obstetrical wards. Although he proposed this in 1847, he failed to convince others during his lifetime.

## 4. Conclusions

There is currently compelling evidence for the possible use of melatonin as an adjuvant in the treatment of COVID-19 symptoms. However, additional RCTs are necessary to confirm melatonin’s effects as well as to establish the optimal dosage and safety guidelines for that purpose.

It now appears that COVID-19 may become an endemic disorder such as influenza, reappearing at intervals with different variants such as the current delta version [45]. Thus, a treatment that reduces the intensity of the disorder would be highly useful, even if it is not a specific therapy such as a vaccine. It has also been suggested that it could be useful against other deadly diseases such as Ebola [46]. Detailed studies of the mechanisms by which melatonin controls and modulates inflammatory cytokines and other harmful effects should provide leads to additional useful regulatory factors.

## Data Availability

Not applicable.

## References

[1] Lerner A.B., Case J.D., Takahashi Y., Lee T.H., Mori N. (1958). Isolation of melatonin, a pineal factor that lightens melanocytes. J. Am. Chem. Soc..

[2] Nordlund J.J., Lerner A.B. (1977). The effects of oral melatonin on skin color and on the release of pituitary hormones. J. Clin. Endocrinol. Metab..

[3] Claustrat B., Brun J., Chazot G. (2005). The basic physiology and pathophysiology of melatonin. Sleep Med. Rev..

[4] Hack L.M., Lockley S.W., Arendt J., Skene D.J. (2003). The effects of low-dose 0.5-mg melatonin on the free-running circadian rhythms of blind subjects. J. Biol. Rhythms.

[5] Kvetnoy I., Raikhlin N., Tolkachev V. (1975). Chromatographical detection of melatonin (5-methoxy-N-acetylserotonin) and its biological precursors in enterochromaffine cells. Dokl. Acad. Nauk. SSSR.

[6] Raikhlin N.T., Kvetnoy I.M., Tolkachev V.N. (1975). Melatonin may be synthesised in enterochromaffin cells. Nature.

[7] Grota L.J., Snieckus V., de Silva S.O., Tsui H.W., Holloway W.R., Lewy A.J., Brown G.M. (1981). Radioimmunoassay of melatonin in rat serum. Prog. Neuropsychopharmacol..

[8] Bubenik G.A. (1980). Localization of melatonin in the digestive tract of the rat. Effect of maturation, diurnal variation, melatonin treatment and pinealectomy. Horm. Res..

[9] Bubenik G.A. (2001). Localization, Physiological Significance and Possible Clinical Implication of Gastrointestinal Melatonin. Neurosignals.

[10] Acuña-Castroviejo D., Escames G., Venegas C., Díaz-Casado M.E., Lima-Cabello E., López L.C., Rosales-Corral S., Tan D.X., Reiter R.J. (2014). Extrapineal melatonin: Sources, regulation, and potential functions. Cell. Mol. Life Sci..

[11] Slominski A.T., Hardeland R., Zmijewski M.A., Slominski R.M., Reiter R.J., Paus R. (2018). Melatonin: A Cutaneous Perspective on its Production, Metabolism, and Functions. J. Invest. Dermatol..

[12] Kobayashi H., Kromminga A., Dunlop T.W., Tychsen B., Conrad F., Suzuki N., Memezawa A., Bettermann A., Aiba S., Carlberg C. (2005). A role of melatonin in neuroectodermal-mesodermal interactions: The hair follicle synthesizes melatonin and expresses functional melatonin receptors. FASEB J..

[13] Maldonado M.D., Mora-Santos M., Naji L., Carrascosa-Salmoral M.P., Naranjo M.C., Calvo J.R. (2010). Evidence of melatonin synthesis and release by mast cells. Possible modulatory role on inflammation. Pharmacol. Res..

[14] Venegas C., García J.A., Escames G., Ortiz F., López A., Doerrier C., García-Corzo L., López L.C., Reiter R.J., Acuña-Castroviejo D. (2012). Extrapineal melatonin: Analysis of its subcellular distribution and daily fluctuations. J. Pineal Res..

[15] Xia Y., Chen S., Zeng S., Zhao Y., Zhu C., Deng B., Zhu G., Yin Y., Wang W., Hardeland R. (2019). Melatonin in macrophage biology: Current understanding and future perspectives. J. Pineal Res..

[16] Hardeland R., Poeggler B. (2003). Non-vertebrate melatonin. J. Pineal Res..

[17] Hardeland R. (2016). Melatonin in plants – Diversity of levels and multiplicity of functions. Front. Plant Sci..

[18] Shukla M., Chinchalongporn V., Govitrapong P., Reiter R.J. (2019). The role of melatonin in targeting cell signaling pathways in neurodegeneration. Ann. N. Y. Acad. Sci..

[19] Hardeland R. (2017). Melatonin and the electron transport chain. Cell. Mol. Life Sci..

[20] Tan D.-X., Reiter R.J. (2019). Mitochondria: The birth place, battle ground and the site of melatonin metabolism in cells. Melatonin Res..

[21] Tan D., Hardeland R. (2020). Potential utility of melatonin in deadly infectious diseases related to the overreaction of innate immune response and destructive inflammation: Focus on COVID-19. Melatonin Res..

[22] Xu X., Wang G., Ai L., Shi J., Zhang J., Chen Y.X. (2018). Melatonin suppresses TLR9-triggered proinflammatory cytokine production in macrophages by inhibiting ERK1/2 and AKT activation. Sci. Rep..

[23] Hardeland R. (2018). Melatonin and inflammation—Story of a double-edged blade. J. Pineal Res..

[24] Cardinali D.P., Brown G.M., Pandi-Perumal S.R. (2020). Can Melatonin Be a Potential “Silver Bullet” in Treating COVID-19 Patients?. Diseases.

[25] Reynolds J.L., Dubocovich M.L. (2021). Melatonin multifaceted pharmacological actions on melatonin receptors converging to abrogate COVID-19. J. Pineal Res..

[26] Zhai X., Wang N., Jiao H., Zhang J., Li C., Ren W., Reiter R.J., Su S. (2021). Melatonin and other indoles show antiviral activities against swine Coronaviruses in vitro at pharmacological concentrations. J. Pineal Res..

[27] Brusco L.I., Cruz P., Cangas A.V., Rojas C.G., Vigo D.E., Cardinali D.P. (2021). Efficacy of melatonin in non-intensive care unit patients with COVID-19 pneumonia and sleep dysregulation. Melatonin Res..

[28] Hardeland R., Cardinali D.P., Srinivasan V., Spence D.W., Brown G.M., Pandi-Perumal S.R. (2011). Melatonin---A pleiotropic, orchestrating regulator molecule. Prog. Neurobiol..

[29] Emens J., Eastman C. (2017). Diagnosis and Treatment of Non-24-h Sleep-Wake Disorder in the Blind. Drugs.

[30] Auld F., Maschauer E.L., Morrison I., Skene D.J., Riha R.L. (2017). Evidence for the efficacy of melatonin in the treatment of primary adult sleep disorders. Sleep Med. Rev..

[31] Gobbi G., Comai S. (2019). Differential function of melatonin MT1 and MT2 receptors in REM and NREM sleep. Front. Endocrinol..

[32] Haspel J.A., Anafi R., Brown M.K., Cermakian N., Depner C., Desplats P., Gelman A.E., Haack M., Jelic S., Kim B.S. (2020). Perfect timing: Circadian rhythms, sleep, and immunity — An NIH workshop summary. JCI Insight.

[33] Reiter R.J., Tan D.X., Kim S.J., Cruz M.H.C. (2014). Delivery of pineal melatonin to the brain and SCN: Role of canaliculi, cerebrospinal fluid, tanycytes and Virchow–Robin perivascular spaces. Brain Struct. Funct..

[34] Lewandowska K., Małkiewicz M.A., Siemiński M., Cubała W.J., Winklewski P.J., Mędrzycka-Dąbrowska W.A. (2020). The role of melatonin and melatonin receptor agonist in the prevention of sleep disturbances and delirium in intensive care unit – a clinical review. Sleep Med..

[35] Cardinali D.P., Brown G.M., Reiter R.J., Pandi S.R. (2020). Elderly as a High-risk Group during COVID-19 Pandemic: Effect of Circadian Misalignment, Sleep Dysregulation and Melatonin Administration. Sleep Vigil..

[36] Castillo R.R., Quizon G.R.A., Juco M.J.M., Roman A.D.E., de Leon D.G., Punzalan F.E.R., Guingon R.B.L., Morales D.D., Tan D.-X., Reiter R.J. (2020). Melatonin as adjuvant treatment for coronavirus disease 2019 pneumonia patients requiring hospitalization (MAC-19 PRO): A case series. Melatonin Res..

[37] Reiter R.J., Abreu-Gonzalez P., Marik P.E., Dominguez-Rodriguez A. (2020). Therapeutic Algorithm for Use of Melatonin in Patients With COVID-19. Front. Med..

[38] Farnoosh G., Akbariqomi M., Badri T., Bagheri M., Izadi M., Saeedi-Boroujeni A., Rezaie E., Ghaleh H.E.G., Aghamollaei H., Fasihi-ramandi M. (2021). Efficacy of a Low Dose of Melatonin as an Adjunctive Therapy in Hospitalized Patients with COVID-19: A Randomized, Double-blind Clinical Trial. Arch. Med Res..

[39] Hosseini A., Esmaeili Gouvarchin Ghaleh H., Aghamollaei H., Fasihi Ramandi M., Alishiri G., Shahriary A., Hassanpour K., Tat M., Farnoosh G. (2021). Evaluation of Th1 and Th2 mediated cellular and humoral immunity in patients with COVID-19 following the use of melatonin as an adjunctive treatment. Eur. J. Pharmacol..

[40] Chavarría A.P., Vázquez R.R.V., Cherit J.G.D., Bello H.H., Suastegui H.C., Moreno-Castañeda L., Alanís Estrada G., Hernández F., González-Marcos O., Saucedo-Orozco H. (2021). Antioxidants and pentoxifylline as coadjuvant measures to standard therapy to improve prognosis of patients with pneumonia by COVID-19. Comput. Struct. Biotechnol. J..

[41] Ramlall V., Zucker J., Tatonetti N. (2020). Melatonin is significantly associated with survival of intubated COVID-19 patients. medRxiv Prepr. Serv. Heal. Sci..

[42] Sánchez-González M.Á., Mahíllo-Fernández I., Villar-Álvarez F., Llanos L. (2021). What if melatonin could help COVID-19 severe patients?. Clin. Sleep Med..

[43] Eriksson E., Humble M., Pohl R., Gershon S. (1990). Serotonin in psychiatric pathophysiology. The Biological Basis of Psychiatric Treatment–Progress in Basic and Clinical Pharmacology v. 3.

[44] De La Vega C.E., Slater S., Ziegler M.G., Lake C.R., Murphy D.L. (1977). Reduction in plasma norepinephrine during fenfluramine treatment. Clin. Pharmacol. Ther..

[45] Van Doorn H.R. (2021). The epidemiology of emerging infectious diseases and pandemics. Medicine.

[46] Reiter R.J., Ma Q., Sharma R. (2020). Treatment of Ebola and other infectious diseases: Melatonin “goes viral”. Melatonin Res..

